# Das Landesprogramm Bildung und Gesundheit NRW (BuG) – Nachhaltige Entwicklung und Etablierung der *Guten Gesunden Schule* im Bundesland Nordrhein-Westfalen

**DOI:** 10.1007/s00103-022-03552-9

**Published:** 2022-06-13

**Authors:** Marleen Opitz

**Affiliations:** Landesprogramm Bildung und Gesundheit Nordrhein-Westfalen, Landeszentrum Gesundheit Nordrhein-Westfalen, Gesundheitscampus 10, 44801 Bochum, Deutschland

**Keywords:** Schulentwicklung, Gesundheitsförderung, Prävention, Gesundheitskompetenz, Partizipation, School development, Promotion of good health, Prevention, Health expertise, Participation

## Abstract

Mit dem Landesprogramm Bildung und Gesundheit Nordrhein-Westfalen (BuG NRW) bilden alle daran beteiligten Träger eine Verantwortungspartnerschaft zur Förderung der *Guten Gesunden Schule* und zur Umsetzung des Präventionsgesetzes in Nordrhein-Westfalen. Das Ministerium für Schule und Bildung (MSB NRW), das Ministerium für Arbeit, Gesundheit und Soziales (MAGS NRW), die gesetzlichen Krankenkassen/-verbände (GKV NRW) und die Unfallkasse (UK NRW) kooperieren für die Förderung der integrierten Gesundheits- und Qualitätsentwicklung sowie Unterstützung der Gesundheitsförderung und Prävention in Schulen. Zentrale Elemente der Public-Health-Forschung, wie beispielsweise Partizipation (aller Beteiligten) und Empowerment, sind Bestandteil des Gesamtkonzeptes.

Gemeinsames Leitziel der Programmträger ist es, Gesundheits- und Bildungschancen von Kindern und Jugendlichen sowie das Wohlbefinden und die Leistungsfähigkeit aller Beteiligten in der Lebenswelt Schule gerecht und nachhaltig zu verbessern. Dies gelingt für die Programmschulen mit der fachkompetenten Beratung durch persönliche Ansprechpartner*innen, vielfältige Unterstützung, regelmäßige Evaluation sowie die Finanzierung nachhaltiger Schulentwicklungsmaßnahmen zur Verankerung von Gesundheitsförderung und Prävention. Die Gesundheitsqualität und Gesundheitskompetenz aller Beteiligten in der Lebenswelt Schule können dadurch verbessert werden.

Dieser Beitrag gibt Einblicke in das langjährige, anerkannte Schulentwicklungsprogramm, dessen Fortschreibung durch die Kooperationspartner aktuell für weitere 5 Jahre geplant ist. Die neueste Evaluation von BuG NRW hat u. a. gezeigt, wie sich durch implementierte Strukturen, eine aktive Vernetzung und individuelle Unterstützungsangebote in NRW gesundheitsförderliche Schulentwicklung langfristig weiterentwickeln und etablieren kann.

## Abstract (en)

With the “Education and Health NRW” programme, all of the participating sponsors have formed a partnership of shared responsibility for the funding of the *Good Healthy School* and the implementation of the Prevention Act in North Rhine-Westphalia (NRW). The State Ministries of Schools and Education (MSB NRW) and of Labour, Health and Welfare (MAGS NRW), along with the State Statutory Health Insurers (GKV NRW) and Statutory Accident Insurance for the public sector in North Rhine-Westphalia (UK NRW), cooperate in advocating integrated health and quality development and in supporting the promotion of good health and prevention in schools. Central elements of public health research such as the participation (of everyone involved) and empowerment are components of the overall concept.

The joint key objective of the programme sponsors is adequately and sustainably improving health and educational opportunities for children and young people as well as the performance capabilities of everyone involved in the school environment. This is achieved for the schools participating in the programme through expert advice with personal contact persons, wide-ranging support, evaluation on a regular basis, and the financing of sustainable school development measures aimed at firmly establishing the promotion of good health and prevention. This increases the quality of health and health expertise for everyone participating in the day-to-day school environment.

This article provides insights into the recognized school development programme, which has been prolonged by the cooperation partners for an additional five years. One of the things revealed by the latest evaluation of BuG NRW is how implemented structures, active networking, and individual support services in North Rhine-Westphalia can advance and establish long-term school development that promotes good health.

## Einleitung

Gesundheit und Wohlbefinden sind wesentliche Faktoren für die Entwicklung von Persönlichkeit und Fähigkeiten sowie wichtige Voraussetzungen für Lernen und Arbeiten [1]. Sie bestimmen außerdem die Lebensqualität von Kindern, Jugendlichen und Erwachsenen maßgeblich [2]. Schule als Lebenswelt von Kindern, Jugendlichen, dort tätigen Erwachsenen und Eltern hat daher auch die Aufgabe, die Gesundheit aller Beteiligten zu stärken und zu fördern, indem sie ein Umfeld schafft, das sowohl Leistungsfähigkeit und Motivation als auch die Gesundheit fördert [3].

In der schulischen Gesundheitsförderung geht man auf dem Bundesgebiet von unterschiedlichen Strategien für deren Umsetzung aus [4]. So stellt die *verhaltensbasierte Gesundheitsförderung und Prävention in Schule* einen Ansatz dar, der eher auf die individuellen Faktoren der Gesundheit einzelner Personengruppen gerichtet ist [4]. Die *gesundheitsfördernde Schule* hingegen nimmt als komplexerer, durchaus anerkannter, ganzheitlicher Ansatz [4] den Schulentwicklungsprozess mit zentralen Grundprinzipien der Gesundheitsförderung in den Fokus. Dieser Ansatz ist der dritten Strategie, der Leitidee der *Guten Gesunden Schule* durchaus immanent. Jene weist zudem eine Verschränkung von Bildung und Gesundheit auf [4]. Sie beruht auf der Grundannahme, dass zwischen Gesundheitsförderung und Prävention auf der einen Seite und schulischer Qualitätsentwicklung auf der anderen Seite ein intensiver wechselseitiger Zusammenhang besteht [3, 5] – dem spezifischen Verständnis der Wechselbeziehung von Gesundheit und Bildung [3, 6]. Diese Kohärenz ist in der Public-Health-Forschung mehrfach belegt [7] und wird zum Beispiel aktuell durch die Studie zum Nachweis des Zusammenhangs von körperlicher Fitness, Konzentration und gesundheitsbezogener Lebensqualität der Fakultät für Sport- und Gesundheitswissenschaften der Technischen Universität (TU) München erneut belegt [8].

Auf der Leitidee der *Guten Gesunden Schule *fußt das Landesprogramm „Bildung und Gesundheit Nordrhein-Westfalen“ (nachfolgend BuG NRW). So haben sich im Bundesland Nordrhein-Westfalen das Ministerium für Schule und Bildung NRW (MSB), das Ministerium für Arbeit, Gesundheit und Soziales NRW (MAGS), alle gesetzlichen Krankenkassen (GKV) sowie die Unfallkasse NRW (UK) zu einer Verantwortungspartnerschaft bekannt. Intention ist, die Gesundheitsförderung in Schulen systematisch zu integrieren und Schulentwicklung sowie die Stärkung der Gesundheitskompetenzen und -chancen mit der Leitidee der* Guten Gesunden Schule* [5] im Land Nordrhein-Westfalen nachhaltig zu etablieren. Vor dem Hintergrund des gesetzlichen Präventionsauftrages und der Landesrahmenvereinbarung zur Umsetzung der nationalen Präventionsstrategie gemäß § 20f Fünftes Buch Sozialgesetzbuch (SGB V) im Land Nordrhein-Westfalen [9] bildet BuG NRW ein Dach für gemeinsame Initiativen der Programmträger.

In diesem Beitrag werden die Programmkonzeption, die Programmziele, die implementierten Strukturen und Erkenntnisse aus der aktuellen Programmevaluation, aber auch die wichtige Vernetzung mit Kooperationspartner*innen von BuG NRW vorgestellt. Ein Ausblick auf die nächste Programmphase setzt den Fokus auf die Auswirkungen der Coronapandemie und daraus resultierende Anpassungen der Arbeit des Programms.

## Programmkonzeption

Nach 3 erfolgreichen Konzeptphasen (2009–2022) haben sich die Träger des Landesprogramms BuG NRW entschlossen, diese kohärente Zielrichtung für weitere 5 Jahre fortzuschreiben.

„Der Ansatz der integrierten Gesundheits- und Qualitätsentwicklung mit dem Leitmotiv ‚*Gute Gesunde Schule‘* bildet die theoretische Grundlage des Landesprogramms und beschreibt seine grundsätzliche Ausrichtung“ [3]. Sie erzeugen gleichermaßen positive Wirkungen auf die Umsetzung des Bildungs- und Erziehungsauftrags von Schulen. „Prävention und Gesundheitsförderung sind integrale Bestandteile von Schulentwicklung. Sie stellen keine Zusatzaufgaben der Schulen dar, sondern gehören zum Kern eines jeden Schulentwicklungsprozesses“ [10]. Sie stellen einen „lohnenden Beitrag zu mehr Schulqualität und Schulwirksamkeit“ [11] dar. Dadurch wird die grundsätzliche Haltung aller am Schulleben Beteiligten geprägt.

„Eine *Gute Gesunde Schule* ist demzufolge eine Schule, die Unterricht und Erziehung, Lehren und Lernen, Führung und Management sowie Schulkultur und Schulklima durch geeignete Maßnahmen gesundheitsförderlich gestaltet und so die Bildungsqualität insgesamt verbessert. Gleichzeitig verwirklicht sie die spezifischen Gesundheitsbildungsziele, die zu ihrem Bildungs- und Erziehungsauftrag gehören. Darüber hinaus nutzt die Schule auch das präventive und gesundheitsförderliche Potential originär pädagogischer Maßnahmen (z. B. der individuellen Förderung und der Inklusion) für die Erhöhung der Gesundheitsqualität der schulischen Akteur*innen und des Systems Schule als Ganzem“ [3].

Grundlage des Landesprogramms sind Unterstützungsleistungen, die auf der Basis von Evaluationsergebnissen eingeleitet und weiterentwickelt werden und zur Herstellung von mehr Chancengerechtigkeit im Gesundheits- und Bildungsbereich führen. Darüber hinaus werden insbesondere die Partizipation aller Beteiligten in der Lebenswelt sowie Aktivitäten, die das Empowerment dieser Beteiligten fördern, gelebt.

Bei der Umsetzung des Landesprogramms werden auf allen Ebenen die salutogenen Prinzipien [12] der Verstehbarkeit, der Sinnhaftigkeit und der Handhabbarkeit beachtet. Das bedeutet unter anderem, dass Transparenz, Motivation sowie ein schonender und effizienter Umgang mit Ressourcen handlungsleitend sind [3].

## Programmziele

„Gemeinsames Leitziel der Programmträger ist es, Gesundheits- und Bildungschancen von Kindern und Jugendlichen sowie das Wohlbefinden und die Leistungsfähigkeit aller in der Lebenswelt Schule Beteiligten nachhaltig zu verbessern“ [3].

Folgende Bildungs- und Gesundheitsziele tragen zur Erreichung des Leitziels bei:

- Förderung der Gesundheits- und Lebenskompetenz: wie beispielsweise Gesundheitseinstellungen, Gesundheitsbewusstsein, Gesundheitsverhalten (personale und soziale Fähigkeiten) sowie Gesundheitserleben der Personen (Verhaltensprävention; [3]),
- Verbesserung der gesundheitsrelevanten Rahmenbedingungen für alle Beteiligten in der Lebenswelt Schule (Verhältnisprävention; [3]),
- Verbesserung der Bildungsqualität in den Schulen [3],
- Verbesserung der Integration von Maßnahmen und Themen der Gesundheitsförderung und Prävention in Bildung, Wissenschaft und vor allem in die Schul- und Bildungspolitik als Beitrag für mehr Teilhabe und Chancengerechtigkeit [3, 13],
- Unterstützung der Programmschulen in ihrer Schulentwicklung und bei der Bewältigung der Folgen besonderer Belastungen vor dem Hintergrund besonderer gesundheitlicher und gesellschaftlicher Krisensituationen (z. B. Coronapandemie) und damit assoziierter zusätzlicher psychosozialer Belastungen.

## Gelebte Verantwortungspartnerschaft – ein Rückblick

Ursprünglich entwickelte sich aus einem Modellversuch der BLK (Bund-Länder-Kommission) „OPUS – Offenes Partizipationsnetz und Schulgesundheit, Gesundheitsförderung durch vernetztes Lernen“ (1997 bis 2000) das „OPUS NRW – Netzwerk Bildung und Gesundheit“ als ein Landesprogramm von 2000 bis 2007. Aus diesem Vorgängerprogramm „OPUS NRW“ ist durch die Entwicklung der Leitidee der *Guten Gesunden Schule* [5] und neue Partner*innen im Jahr 2009 das jetzige Landesprogramm Bildung und Gesundheit NRW (BuG NRW) als gemeinsames Programm der Landesregierung Nordrhein-Westfalens vertreten durch die folgenden Träger hervorgegangen:

- Das *Ministerium für Schule und Bildung des Landes Nordrhein-Westfalen (MSB NRW)* misst der Gesundheitsförderung zur Erhöhung der Bildungsqualität eine wesentliche Bedeutung bei und möchte diese nachhaltig mit Schul- und Unterrichtsentwicklung verbinden. Es folgt dabei den Qualitätsaussagen, die im § 2 (Abs. 4, 5 u. 6) Schulgesetz NRW, im Referenzrahmen Schulqualität NRW [14] und in den „Empfehlungen zur Prävention und Gesundheitsförderung in der Schule“ der KMK (Kultusministerkonferenz) vom 15.11.2012 formuliert wurden [10]. Basis für das schulische Handeln ist das umfassende Verständnis von Gesundheit, welches im Referenzrahmen Schulqualität NRW neben Fragen der Gesundheitsbildung und Prävention auch psychische Gesundheit umfasst.
- Das *Ministerium für Arbeit, Gesundheit und Soziales (MAGS NRW)*, unterstützt durch das Landeszentrum Gesundheit Nordrhein-Westfalen (LZG.NRW), begleitet das Landesprogramm als das für Gesundheit und die Landesrahmenvereinbarung zur Umsetzung des Präventionsgesetzes [15] und die Landesinitiative „Gesundheitsförderung und Prävention“ [16] federführende Ressort der Landesregierung sowie als fachliche Leitstelle für den Öffentlichen Gesundheitsdienst.
- Die Trägerschaft der *Unfallkasse NRW (UK NRW)* und die damit verbundene Unterstützung der Mitgliedsschulen erfolgt im Rahmen der gesetzlichen Regelungen des § 14 des Siebten Buchs Sozialgesetzbuch (SGB VII). Danach hat die Unfallkasse NRW laut Absatz 1: „… mit allen geeigneten Mitteln für die Verhütung von Arbeitsunfällen, Berufskrankheiten und arbeitsbedingten Gesundheitsgefahren sowie für eine wirksame Erste Hilfe zu sorgen“.
- Die Trägerschaft der *gesetzlichen Krankenkassen/-verbände NRW (GKV)* begründet sich im gesetzlichen Präventionsauftrag nach §§ 20, 20a und 20b SGB V in Verbindung mit dem Leitfaden Prävention des GKV-Spitzenverbandes in der jeweils aktuellen Fassung [17]. Ziel ist es demnach, durch Leistungen der Primärprävention und Gesundheitsförderung in der Lebenswelt Schule Ressourcen für ein gesundes Leben zu fördern und Gesundheitskompetenzen zu verbessern und insbesondere einen Beitrag zur Verminderung sozial bedingter Ungleichheit von Gesundheitschancen zu erbringen sowie die Schaffung bzw. die Stärkung von Strukturen, welche einen Beitrag zu gesundheitsförderlichen Lebens‑, Lern- und Arbeitsbedingungen leisten.

## Implementierte Strukturen – ein Einblick

### Struktur der Zusammenarbeit

Die strategische und inhaltliche Steuerung und Gestaltung des Landesprogramms wird von einer Steuerungsgruppe wahrgenommen, in welcher alle Träger des Programms stimmberechtigt vertreten sind. Für die Sicherstellung der Beratung, Fortbildung und Netzwerkarbeit auf Regierungsbezirksebene sind Dezernent*innen zuständig. Diese benennen Lehrer*innen als BuG-Koordinator*innen, die die operative Arbeit im Landesprogramm leisten.

Die BuG-Koordinator*innen sind auf der Ebene der 5 Bezirksregierungen in je einem Bezirksteam organisiert, welche in Absprache mit der zuständigen Dezernentin bzw. dem Dezernenten die Arbeitsplanung mit Zielen und Maßnahmen für den jeweiligen Regierungsbezirk festlegen. Die Koordinator*innen werden durch fortlaufende Qualifizierung und Fortbildung befähigt und unterstützt. Insbesondere die Entwicklungsprozesse der jeweiligen Schule beratend zu begleiten und zu fördern, Netzwerke und Kooperationen aufzubauen, zu betreuen und weiterzuentwickeln, gehören zu ihren Aufgaben [3]. Für diese Tätigkeiten werden die Koordinator*innen als Trägerbeitrag des MSB NRW für das Programm abgeordnet.

**Figure 1:**
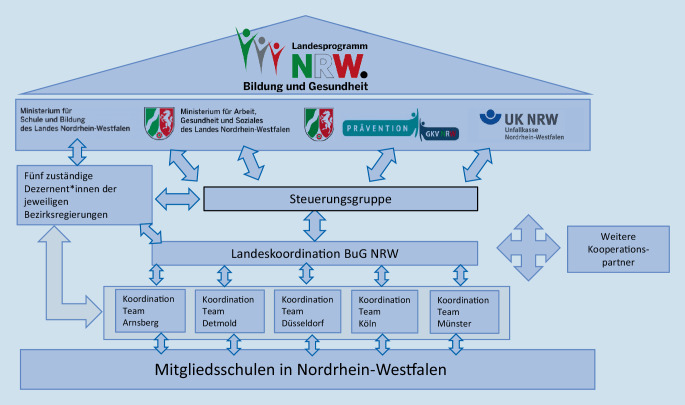

Die Koordination der Arbeit zwischen den Regierungsbezirken und die überregionale Vernetzung sowie die Geschäftsführung des Programms obliegen der Landeskoordination. In Abb. 1 werden die verschiedenen Arbeitsebenen und Gremien in ihrer Beziehung zueinander dargestellt.

### Die Mitgliedschaft

Jede Schule im Land NRW kann Mitglied im Landesprogramm werden und sich durch eine Kooperationsvereinbarung – mittels Schulkonferenzbeschluss als Bekenntnis der gesamten Schulgemeinde – der Leitidee der *Guten Gesunden Schule* verschreiben [3].

Eine integrierte Gesundheits- und Qualitätsentwicklung in Schulen baut auf der aktiven Mitwirkung der Beteiligten auf, die dadurch den Prozess der Veränderung ihrer Bildungseinrichtung bewusst mitgestalten [3]. Nur durch deren Aktivierung und die Mobilisierung eigener Kräfte (Empowerment) ist eine nachhaltige Veränderung zu erreichen.

Im Rahmen der Mitgliedschaft durchläuft eine jede Schule insgesamt 3 festgelegte Programmphasen der Schulentwicklung:

- *Phase 1:* Maßnahmen zum Aufbau von gesundheitsförderlichen Strukturen und Prozessen,
- *Phase 2:* Maßnahmen zur Schul- und Unterrichtsentwicklung zu eigenen gesundheitsförderlichen Schwerpunkten,
- *Phase 3: *Maßnahmen zur Entwicklung eines Qualitätsmanagements.

**Figure 2:**
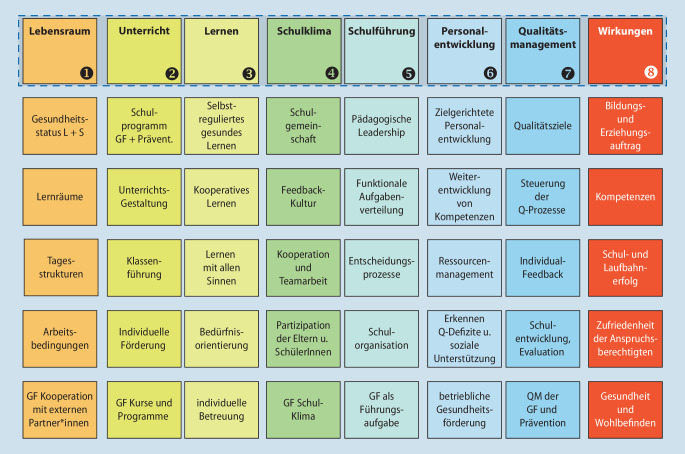

In Phase 1 schafft die Schule gesundheitsförderliche sowie geeignete partizipative Strukturen [3] und integriert diese fest in ihr Leitbild/Schulprogramm. Zudem wird durch die BuG-Standortbestimmung datengestützt die Schul- und Unterrichtsentwicklung ausgerichtet. Die Durchführung und Auswertung der Evaluationen des Landesprogramms BuG NRW erfolgen mit dem Institut für Arbeit und Gesundheit (IAG) der Deutschen Gesetzlichen Unfallversicherung (DGUV). Diese regelmäßige, formative Evaluation basiert auf dem Wirkungsmodell des IQES-Qualitätstableaus (Instrumente der Qualitätsentwicklung und Selbstevaluation an Schulen; [18]) und liegt dem Konzept der *Guten Gesunden Schule *[5] zugrunde (Abb. 2). Anhand der von IQES entwickelten Qualitätsbereiche und Dimensionen kann das System Schule diese BuG-Standortbestimmung vornehmen und infolge daraus Entwicklungen ableiten. In der ersten Phase vernetzt sich die Mitgliedsschule bereits aktiv mit anderen BuG-Schulen und wird durch programmrelevante Fortbildungen zu gesundheitsförderlichen Schwerpunkten sowie z. B. schulformbezogenen Netzwerkveranstaltungen unterstützt.

Programmschulen, welche die erste Phase erfolgreich abgeschlossen haben, setzen sich mithilfe der durch die BuG-Standortbestimmung gewonnenen Erkenntnisse eigene gesundheitsförderliche Schwerpunkte in der Schul- und Unterrichtsentwicklung (Phase 2) und entwickeln dabei nach und nach ein Qualitätsmanagement (Phase 3), das in langfristigen Schulentwicklungsplänen dokumentiert wird. Der Übergang von Phase 2 zu Phase 3 ist fließend angelegt. Während dieser Phasen werden Vernetzungen u. a. mit geeigneten weiteren Einrichtungen und außerschulischen Partner*innen weiter ausgebaut und intensiviert. Das Zusammenwirken soll nachhaltige Synergieeffekte auf allen Ebenen schaffen. Das Schulprogramm dient dabei als dynamisches Arbeitsinstrument und wird fortlaufend weiterentwickelt.

Von besonderer Bedeutung und hoher Wertschätzung bei den knapp 350 Mitgliedsschulen ist in allen Phasen der Mitgliedschaft die fachkompetente Begleitung und Beratung der für jede Programmschule explizit zuständigen, qualifizierten Koordinator*innen. Aber auch die Möglichkeit der finanziellen Förderung bei nachhaltig angelegten Schulentwicklungsvorhaben ist für die Schulen eine wichtige Unterstützung. Der Inhalt der onlinebasierten Antragstellung passt auf die 3 Phasen der Mitgliedschaft einer BuG-Schule.

Die Verknüpfung von Verhältnis- und Verhaltensprävention sowie die nachhaltige Wirksamkeit einer geplanten Maßnahme sind grundlegende Voraussetzungen für die finanzielle Förderung durch das Landesprogramm BuG NRW. Die Finanzierungsmöglichkeiten unterliegen den Kriterien des jeweils aktuellen Leitfadens Prävention der GKV [17]. Zentraler Bestandteil der Qualitätssicherung aller Maßnahmen ist der Public Health Action Cycle (PHAC), wie er von Rosenbrock weiterentwickelt wurde [19].

Durch die Zusammenarbeit des Programms mit dem Landeszentrum Gesundheit NRW wurde zudem ein ergänzendes Modul entwickelt und evaluiert [20]. In diesem wird eine partizipative Gesundheitsbildung und -förderung qualitätsgesichert und nachhaltig im Setting Schule integriert: Schüler*innen werden gemeinsam mit Lehrer*innen zu gesundheitsförderlichen Beteiligungsteams (B-Teams) qualifiziert. Dabei berücksichtigen die B‑Teams die Interessen von Schüler*innen und Lehrpersonen gleichermaßen im Schulsystem und werden somit fest in einen kooperativen und gesundheitsbezogenen Schulalltag implementiert.

Das Landesprogramm bietet flankierend über die Homepage eine Datenbank für weitere wertvolle, aktuelle Unterstützungsangebote der Träger sowie weiterer Kooperationspartner von BuG NRW allen Schulen frei zugänglich an. Diese Angebote sind nach Zielgruppe, Themenschwerpunkt und Anbieter filterbar und für die Schulen grundsätzlich kostenfrei.

## Vielfältige Vernetzung mit weiteren Kooperationspartnern

Nicht nur für die qualitative Arbeit des Programms sind Kooperationen wegweisend, sondern auch für dessen langfristig anzustrebendes Wachstum. Auf 2 Ebenen lassen sich die Programmvernetzungen beschreiben: auf der Programmebene und auf der institutionellen Ebene.

Es wird auf der Ebene der Programmschulen eine intensive Vernetzung untereinander durch schulformbezogene oder regionale Veranstaltungen fokussiert. Durch die pandemiebedingt notwendige Umstellung der Veranstaltungsdurchführung auf onlinebasierte Formate hat sich zudem eine gewinnbringende Vernetzung auch über die Grenzen der 5 Bezirksregierungen hinaus für die Schulen entwickelt.

Auf der institutionellen Ebene sind vielfältige Kooperationen des Landesprogramms mit landesweiten Akteur*innen kontinuierlich gewachsen. So sind im Rahmen der Landesinitiative „Gesundheitsförderung und Prävention“ als Entschließung der Landesgesundheitskonferenz Nordrhein-Westfalen [16] Strukturen und Gremien als wichtige Vernetzungen etabliert. In dieser bietet beispielsweise die Ginko-Stiftung für Prävention als Träger der Landesfachstelle Suchtprävention NRW wertvolle Expertise. Sie entwickelt für die Schulen in NRW bedarfsgerecht attraktive Projekte und Unterstützungsmöglichkeiten zur Suchtprävention. Das aktuelle Modellprojekt „Suchtprävention an berufsbildenden Schulen“ in Kooperation mit BuG NRW (finanziert durch die GKV sowie UK NRW) ist ein Beispiel für diese erfolgreiche Zusammenarbeit. Es stehen Maßnahmen zur Suchtvorbeugung von Tabak‑, Alkohol- und Cannabiskonsum im Mittelpunkt des Projektes, welche die nachweislich sinnstiftende Strategie der Verhältnis- und Verhaltensprävention in diesem Handlungsfeld und den derzeitig positiven Trend [21] weiter verstärken sollen.

Unter Federführung des MSB NRW konnte eine weitere Kooperation des Landesprogramms mit dem Vorhaben GigS (Ganztagsberufsschule in der gesunden Schule; [22]) als einer alternativen Organisationsform für die Berufsschule mit integrierter Gesundheitsförderung vereinbart werden.

Zudem verbindet eine langjährige Partnerschaft BuG NRW mit den Akteur*innen des Schweizer Portals zur Schul- und Unterrichtsentwicklung IQESonline (Digitale Arbeits- und Lernplattform für Schule und Unterricht, Winterthur, Schweiz). Durch eine umfassende finanzielle Förderung durch die Träger des Landesprogramms wird den Mitgliedsschulen der Zugang zu Evaluations- und Feedbackinstrumenten dieses Portals ermöglicht und dadurch auch vielfältige Methoden zum digitalen, kooperativen Lernen und zur salutogenen Unterrichtsentwicklung zur Verfügung gestellt. Über IQESonline besteht zudem ein Kontakt zu vergleichbaren schulischen Netzwerken in anderen deutschsprachigen europäischen Regionen.

Eine weitere Vernetzung findet über die Präsenz des Landesprogramms in verschiedenen Arbeitsgremien auf Landesebene oder auch durch die Zusammenarbeit mit der Fachgruppe der Kinder- und Jugendgesundheit im LZG.NRW statt.

### Programmevaluation auf Grundlage des IQES-Tableaus

Die datengestützte Schulentwicklung und Selbstevaluation sind ein wesentliches Qualitätsmerkmal der *Guten Gesunden Schule *[18]. Eine kontinuierliche und wissenschaftlich fundierte Evaluation des Landesprogramms BuG NRW wurde in den Jahren 2013 bis 2018 durch die Abteilung für Bildungsforschung und Bildungsmanagement der Heinrich-Heine-Universität Düsseldorf durchgeführt und in internen Evaluationsberichten zusammengefasst. Daraus gewonnene Erkenntnisse, wie beispielsweise die Programmwirksamkeit sowie begleitende Rolle der Koordinator*innen oder die vom Landesprogramm eingerichteten Unterstützungsangebote, wurden durch die Schulen als wesentlich bewertet.

Zwischenzeitlich erfolgte im Landesprogramm für die Evaluation die Erarbeitung einer neuen „BuG-Standortbestimmung“ und es wurde die Zusammenarbeit mit dem Institut für Arbeit und Gesundheit (IAG) der Deutschen Gesetzlichen Unfallversicherung (DGUV) aufgenommen. Auf Grundlage eines internen Konzepts kamen auf unterschiedlichen Programmebenen (Lehrkräfte, Schulleitungen, Koordinator*innen, Dezernent*innen und Programmträger) umfassende Fragebögen zum Einsatz. Die Ergebnisse der Evaluation des Landesprogramms der Jahre 2017–2021 bestätigen die Wirksamkeit der konzeptuellen Ausrichtung des Programms (vgl. interner Bericht des IAG der DGUV aus November 2021). „Die Selbsteinschätzung der Schulen zeigt, dass im Vergleich zu vorherigen Evaluationsergebnissen eine leicht steigende Entwicklungstendenz in allen Qualitätsdimensionen zu verzeichnen ist. Besonders positiv werden von Lehrkräften die Dimensionen ‚Unterricht‘, ‚Wirkungen‘ und ‚Schulklima‘ bewertet. Die Qualitätsdimensionen des IQES-Tableaus ‚Schulleitung‘, ‚Schulklima‘ und ‚Qualitätsmanagement‘ korrelieren am stärksten mit dem Bereich ‚Zufriedenheit und Wohlbefinden‘ in der Dimension ‚Wirkungen‘. Je höher die Schulqualität in diesen Dimensionen sowie im Bereich der ‚Kooperationen mit externen Partner*innen‘ eingeschätzt wird, desto positiver wird die Dimension der ‚Wirkungen‘ bewertet“ (vgl. interner Bericht des IAG der DGUV aus November 2021 und Abb. 2). Dieses Ergebnis bestätigt noch einmal den Ansatz der *Guten Gesunden Schule *[5].

In den Ergebnissen wurde auch aufgezeigt, dass insgesamt alle Beteiligten des Landesprogramms (Träger, Dezernent*innen, Koordinator*innen, Schulen) die Bedeutsamkeit des Konzeptes für die schulische Qualitätsentwicklung hervorheben. Dabei werden in der Praxis besonders die individuelle Beratung, die Möglichkeit zur Netzwerkbildung und zum Austausch sowie die weiteren Unterstützungsangebote innerhalb des Landesprogramms als positiv wahrgenommen. Die Programmschulen geben zudem an, dass Maßnahmen wie eine finanzielle Förderung von Schulentwicklungsvorhaben sowie Fortbildungsangebote zukünftig nachhaltige Schulentwicklung in besonderem Maße fördern würden.

Veränderungspotenziale werden zum einen in der Komplexität der Strukturen und Prozesse und zum anderen in der damit verbundenen gemeinsamen Kommunikationskultur verortet (vgl. interner Bericht des IAG der DGUV aus November 2021). Als ein weiterer zentraler Aspekt wird die hohe Belastung der Kollegien durch die Coronapandemie von Programmschulen und BuG-Koordinator*innen betont, welcher durch einen hohen Zeitaufwand für einzelne Prozesse und Strukturen, v. a. im Bereich der Anforderungen an die Programmschulen, verstärkt wird. In diesem Zusammenhang geben die Schulen die Anregung, die Nachwirkungen der Coronapandemie stärker zu berücksichtigen, was mit dem Wunsch einer höheren Flexibilität des Landesprogramms einhergeht (vgl. interner Bericht des IAG der DGUV aus November 2021).

## Zukünftige Weiterentwicklung des Programms

Als zentrale Impulse für die Weiterentwicklung des Landesprogramms werden eine Ausgestaltung von reaktionsfähigen, transparenten und praxisorientierten Prozessen, eine Berücksichtigung der Aspekte „Flexibilität“ und „Kohärenz“ sowie eine Optimierung der Kommunikationsstrukturen zwischen allen Beteiligten des Landesprogramms und die Berücksichtigung der Bedarfe aus der Praxis betont. Intensivierungsbedarf im Bereich der Qualitätsentwicklung liegt weiterhin im gezielten Aufbau eigener schulinterner Evaluationsprozesse, u. a. durch die Nutzung von IQESonline, vor. Zudem bedarf es für die Programmschulen einer weiterführenden Unterstützung, um langfristig angelegte Entwicklungspläne mit Kernelementen des Qualitätsmanagements, wie z. B. eine ressourcenorientierte Personalentwicklung, die Nutzung eines schulinternen Feedback- und Selbstevaluationssystems und die zielführende Steuerung von Qualitätsprozessen, zu erreichen.

Die Coronapandemie hat vor allem die Schulen vor viele und neue Herausforderungen gestellt. Bereits vorhandene sowie auch neu entstehende psychosoziale Belastungen von Kindern, Jugendlichen und Erwachsenen sind nachweisbar [23] und rücken spürbar stärker in den Fokus der Schulentwicklungsarbeit.

Das Programm hat innerhalb der letzten beiden Jahre der Pandemie ein hohes Maß an Flexibilität und Agilität bewiesen. Neben Bürokratieabbau wurde sehr zeitnah eine Umstellung auf digitale Veranstaltungsformate vorgenommen. Weiterhin wurde bedarfsgerecht auch im Sinne der derzeitigen Schwerpunktsetzung der Landesinitiative Gesundheitsförderung und Prävention [16] *„Stärkung der seelischen Gesundheit“* agiert, sodass derzeit eine besondere finanzielle Förderung von Maßnahmen mit dem Fokus auf Stärkung der Resilienz die Schulen bei ihrer Schulentwicklungsarbeit unterstützend erreicht.

In dieser gesundheitlichen und gesellschaftlichen Krise wird jedoch deutlich, dass das bestehende Netzwerk auch tragfähig ist, um besonderen Belastungssituationen begegnen zu können. Die von BuG NRW fokussierte integrierte Gesundheits- und Qualitätsentwicklung ist hilfreich für die Stärkung Einzelner sowie der gesamten Schulgemeinschaft und entfaltet salutogenes Potenzial.

Ausgehend von den Evaluationsergebnissen der noch laufenden Programmphase und vor dem Hintergrund der jeweiligen Satzungen der Programmträger nimmt das Steuerungsgremium für die anstehende Fortführung des Programms in den nächsten 5 Jahren folgende Schwerpunkte in den Blick:

- stetige Weiterentwicklung der „BuG-Standortbestimmung“ für die BuG-Mitgliedsschulen als ein wichtiges Instrument der Selbstevaluation und Standortkennzeichnung der Schulen auf Grundlage des IQES-Tableaus [18],
- Umsetzung der von der programminternen Arbeitsgruppe zur Weiterentwicklung derzeit in der Erarbeitung stehenden Konzeptbausteine. Diese setzen zielführend bei den aktuellen Bedarfen der Programmschulen an.
- Systematische und zielgruppenorientierte Öffentlichkeitsarbeit zur weiteren Verbreitung des BuG-Landesprogramms und zur Information interessierter Schulen,
- Berücksichtigung der Ausgangslagen kleinerer Schulsysteme mit der Fokussierung, insbesondere die Teilnahme von Grundschulen am BuG-Landesprogramm – im Sinne einer möglichst frühen gesundheitsförderlichen Ausrichtung – zu fördern und zu unterstützen, sowie
- neue Kooperationen, welche die Qualität und die Aktualität des Landesprogramms stärken, anvisieren.

Langfristige Auswirkungen der Pandemie gilt es, bei allen schulischen Akteur*innen in der nächsten Programmphase aufmerksam zu beobachten und im Sinne der Kohärenz durch adäquate, gesundheitsförderliche Unterstützungsmöglichkeiten das Programmportfolio bedarfsgerecht auszubauen.

## Fazit

Dieses etablierte Schulentwicklungsprogramm verdeutlicht, dass eine gelebte Verantwortungspartnerschaft mit tragfähigem Netzwerk eine stabile Grundlage schafft, um schulische Gesundheitsförderung und Prävention wirkungsvoll, partizipativ und nachhaltig zu entfalten. Es unterstreicht zudem die Wirksamkeit der Leitidee der *Guten Gesunden Schule *und hat damit Vorzeigecharakter für das Bundesgebiet.
